# The role of the mobile communication devices in the spread of healthcare-associated infections: a systematic review

**DOI:** 10.1186/2047-2994-4-S1-P39

**Published:** 2015-06-16

**Authors:** R Szabó, J Morvai

**Affiliations:** 1 Department of Hospital Epidemiology and Hygiene, National Center for Epidemiology, Budapest, Hungary; 2 School of PH.D. Studies, Semmelweis University, Budapest, Hungary

## Introduction

Mobile communication devices of healthcare workers have an invaluable feature of communication within hospital settings, and they may support aspects of clinical diagnosis and education. However, there may be a potential for contamination with various pathogens.

## Objectives

Our objective was to systematically review the potential role of these devices in the dissemination of pathogens and the effective prevention measures.

## Methods

A comprehensive literature search was performed following the Preferred Reporting Items for Systematic Reviews and Meta-Analyses (PRISMA) guidelines. To identify relevant publications, PubMed, Medline and ScienceDirect were searched for articles published between 1 January 2000 and 1 August 2014 in English language. Appropriate articles were accessed in full text to determine eligibility and extract data by two reviewers. Studies were stratified by pathogens and prevention measures.

## Results

Our search yielded 11,824 titles published after the search period. Finally, 30 articles met our inclusion criteria. Only 8% of healthcare workers routinely cleaned their mobile communication devices which resulting a high rate of contamination (40-100%). Coagulase-negative Staphylococci and *Staphylococcus aureus* were the most common identified bacteria. The most of them was methicillin resistant (10-95.3%).

## Conclusion

This systematic review identified effective interventions to reduce bacterial contamination risks include regularly decontamination of mobile communication devices, hand hygiene and staff education.

## Disclosure of interest

None declared.

